# Growth, Hepatic Enzymatic Activity, and Quality of European Seabass Fed on *Hermetia illucens* and Poultry By-Product Meal in a Commercial Farm

**DOI:** 10.3390/ani14101449

**Published:** 2024-05-13

**Authors:** Lina Fernanda Pulido-Rodriguez, Leonardo Bruni, Giulia Secci, Sara Moutinho, Helena Peres, Tommaso Petochi, Giovanna Marino, Emilio Tibaldi, Giuliana Parisi

**Affiliations:** 1 Department of Agriculture Food Environment and Forestry, University of Florence, Via delle Cascine 5, 50144 Firenze, Italy; linafernanda.pulidorodriguez@unifi.it (L.F.P.-R.); lbruni@iim.csic.es (L.B.); giuliana.parisi@unifi.it (G.P.); 2 Institute of Marine Research (IIM-CSIC), Rúa de Eduardo Cabello 6, 36208 Vigo, Spain; 3 Interdisciplinary Centre of Marine and Environmental Research (CIIMAR), University of Porto, Terminal de Cruzeiros do Porto de Leixões, Av. General Norton de Matos s/n, 4450-208 Matosinhos, Portugal; smoutinho@ciimar.up.pt (S.M.); pereshelena@fc.up.pt (H.P.); 4 Department of Biology, Faculty of Sciences of the University of Porto, Rua do Campo Alegre s/n, Ed. FC4, 4169-007 Porto, Portugal; 5 Department of Sustainable Aquaculture, Italian National Institute for Environmental Protection and Research (ISPRA), Via Vitaliano Brancati 48, 00144 Roma, Italy; tommaso.petochi@isprambiente.it (T.P.); giovanna.marino@isprambiente.it (G.M.); 6 Department of Agri-Food, Environmental and Animal Sciences, University of Udine, Via Sondrio 2, 33100 Udine, Italy; emilio.tibaldi@uniud.it

**Keywords:** alternative protein source, black soldier fly larvae, insect meal, poultry byproduct, *Dicentrarchus labrax*, growth performance, hepatic enzymes, flesh quality

## Abstract

**Simple Summary:**

After a decade of research about the use of insects as fish feed, this paper reports the results of a large-scale trial on European sea bass (*Dicentrarchus labrax*) farmed under commercial conditions. Fish were fed an experimental diet containing 10% *Hermetia illucens* larva meal, 30% poultry by-product meal, and <5.5 g/100 g of feed of marine proteins. The results highlight that fish growth performances and the flesh quality of sea bass fed the experimental diet were similar to those of fish fed a commercial diet containing fish meal and fish oil. This study suggests that a diet rich in plant proteins, in which *H. illucens* and poultry by-products are also included, may be a viable alternative to existing aquafeeds for marine species.

**Abstract:**

Protein meals from insects in combination with poultry by-product meal appear to be promising ingredients for replacing conventional proteins in the diets of carnivorous fish. The present study explored the effects on growth performance, hepatic enzymatic activity, and fillet physical and nutritional characteristics during a 66-day feeding trial performed on European seabass. A total of 3000 fish were distributed into three tanks, where the control group was fed with a commercial diet (CG) and a second group was fed in duplicate with the experimental diet (SSH) containing 10% *Hermetia illucens* larva meal, 30% poultry by-product meal, and <5.5 g/100 g of feed of marine origin proteins. All fish showed good growth performance. Glucose-6-phosphate dehydrogenase, aspartate aminotransferase, and 3-hydroxyacyl-CoA dehydrogenase activities were higher in the SSH group than in the CG group. The fillet fatty acid profile was largely unaffected by diet, except for a few fatty acids. Fish fed the SSH diet had a lower C22:1n-11 content than CG, thus suggesting an increased β-oxidation. The oxidative status of muscle lipids was not affected by the diet. In conclusion, the present study showed that European seabass can be successfully fed the SSH diet for two months in a commercial setting.

## 1. Introduction

Aquaculture’s goal is to achieve sustainable development in all three pillars, that is, to be economically, socially, and environmentally sustainable [1]. The correct formulation of feeds for farmed fish is key to maintaining the sustainable growth of the aquaculture sector within the vision of a circular bioeconomy, without compromising the nutritional quality of the product [2]. Although fishmeal (FM) is an ideal protein source for carnivorous fishes [3], it is a finite resource whose high price and impact on natural ecosystems have led to its use in aquaculture being increasingly reduced and replaced by plant protein sources [4,5]. Despite their high potential for aquaculture development, plant proteins face feed-food competition and have been shown to adversely affect fish growth performance and welfare in carnivorous species [6,7,8].

Protein meals from terrestrial animals, such as black soldier fly (*Hermetia illucens*) meal (HIM) and poultry by-product meal (PBM), appear to be promising replacements for conventional raw materials in diets for carnivorous fishes [9,10]. Both HIM and PBM are not directly intended for human consumption, and their production has a low environmental footprint [11,12]. In addition, the nutritional profile of HIM is similar to that of FM [13,14], and PBM is readily available on the market [15]. Recent results on gilthead seabream (*Sparus aurata*) show that HIM can be included at 11% [16] and 15% [17], partially replacing FM, without compromising fish growth performance, blood biochemistry, or stress parameters; however, the integrity of the intestinal mucosa and submucosa decreased with increasing levels of HIM in the diet [16]. Similarly, the inclusion of up to 19.5% of HIM in FM-based diets for European seabass (*Dicentrarchus labrax*) did not compromise the zootechnical parameters of fish and nutritional characteristics of the fillets and could also contribute to reducing their lipid oxidation [18]. With regard to PBM, several studies demonstrated that it could partially replace FM in feed for juvenile black seabream (*Spondyliosoma cantharus*) [19], gilthead seabream (*Sparus aurata*) [20], and juvenile red porgy (*Pagrus pagrus*) [21] without negative effects on growth performance, survival, or intestinal digestive and absorptive functions.

Although the output of using either HIM or PBM individually is promising, in the frame of sustainable aquaculture intensification, a single protein source is unlikely to meet the essential nutritional requirements of fish and at the same time provide the best quality end-product [10]. During the last five years, the national project “SUstainable fiSH feeds INnovative ingredients–SUSHIN” funded by the AGER2 Network Foundation has evaluated the potential of different unconventional and underused ingredients, tested singly or in combination, as alternative protein sources for aquafeeds, generating new information on the environmental footprint of feeds [12], on fish growth and welfare, and on the nutritional traits of carnivorous fish species economically important for the European aquaculture [22,23,24]. In addition, results obtained under experimental conditions show that feeding gilthead seabream for 18 weeks with diets containing a negligible amount of FM and 40% plant protein replacement by PBM and HIM alone or in combination (30% and 10%, respectively) improved the zootechnical performance of fish and the nutritional characteristics of the fillets, also ensuring physiological well-being and liver health [24,25]. Pleić et al. [26] found that plant-based diets supplemented with HIM in combination with PBM resulted in the highest specific growth rates and lowest feed conversion ratios for European seabass while maintaining the nutritional value of the fillets for human consumption.

Based on the results obtained in other studies under laboratory conditions, the present study aimed to evaluate the effects on growth performance and food quality attributes of European seabass farmed under commercial conditions and fed a diet poor in marine protein, rich in plant protein, and including a combination of HIM and PBM.

## 2. Materials and Methods

### 2.1. Fish Rearing and Diet Formulation

This study was carried out at the Ittica Caldoli fish farm (Foggia, Italy), located near the brackish lagoon of Lesina, in Apulia region. The farm is equipped with a hatchery and a flow-through tank system for the grow-out phase up to commercial size. For the present feeding trial, a total of 3000 mixed-sex European seabass (mean body weight 300 ± 56.3 g) previously raised in an outdoor concrete tank were randomly stocked in indoor fiberglass tanks (24 m^3^ volume) into three replicates at 1000 fish per tank at approximately 12.5 kg/m^3^ and acclimated for one week. Then, a group of 1000 fish was fed as the control group (CG) with a commercial diet (Ecovitae, 4fish s.r.l., Terni, Italy), while the other group of 2000 fish was fed with the experimental diet (SSH) provided by Veronesi feed mills. The feeding trial for the three batches lasted 66 days. The experimental diet was formulated with a combination of HIM (8.1% of the diet, as fed basis) obtained from partially defatted pupae (Table S1), PBM (20.6% of the diet, as fed basis) and a small amount of marine proteins (5.5% of the diet, as fed basis). The ingredients and proximate composition of the CG and SSH diets are shown in Table 1. The ingredients of the CG diet and the fatty acid profile of the CG and SSH diets are reported in the Supplementary Materials (Table S2 and Table S3, respectively). Fish were fed according to the common practice used in the fish farm. Briefly, for the scope of the present feeding trial, a pre-weighed ration per tank was prepared daily and hand-distributed to visual satiety in a single morning meal. At the end of each meal, a visual inspection was carried out to verify no uneaten feed was left in the tank. During the trial, fish were kept under a natural photoperiod at a water temperature of 23.2 ± 0.7 °C, dissolved oxygen of 10.1 ± 0.5 mg/L, and pH between 6.9–7.2. The water drawn from the local underground and used for grow-out had a salinity level of 9.6 ± 1.4 g/L [27].

### 2.2. Fish Sampling

A preliminary sampling was conducted prior to the initiation of experimental feeding (designated as T0, following the acclimation period), during which 15 fish per tank were sampled, euthanized, and subsequently stored at −80 °C for subsequent analyses. Upon completion of the trial period spanning 66 days, 25 fish per tank were sampled, euthanized, and subjected to biometric measurements to assess growth performance. Additionally, 20 fish from the control group (CG) tank and 15 from each treatment tank (SSH) were collected, euthanized, and promptly dispatched in dry ice to the laboratory, where they were stored at −80 °C until further analyses concerning product quality and hepatic enzymatic activity could be conducted. All sampled fish were subjected to a 24 h fasting period prior to sampling and were humanely euthanized using an overdose of tricaine methanesulphonate (MS-222 Pharmaq, AquaVet S.A., Nea Filadelfia, Greece) at a concentration of 300 mg/L [28]. Given the known susceptibility of seabass to stress, particularly during handling [29], no additional sampling was undertaken during the trial to mitigate potential adverse effects on fish health, welfare, feeding behavior, or associated indicators.

### 2.3. Zootechnical Parameters

Body weight (g) and standard and total length (cm) were recorded for each fish sampled. Condition factor (K), specific growth rate (SGR), feed conversion ratio (FCR), and feed intake (FI) were calculated as follows:

K = [(body weight (g)/total length (cm)^3^] × 100 (1)

SGR = [(ln final body weight − ln initial body weight)/days] × 100(2)

FCR = feed administered/weight gain(3)

FI = cumulative feed delivered/number of fish/days(4)

### 2.4. Marketable Characteristics of Fish and Physical Proprieties of Fillets

Ten fish for each tank were thawed overnight at +1 °C. Then, the fish were measured for total length, eviscerated, filleted and the fillets and organs weighed individually to calculate the following parameters:

Fillet Yield, FY (%) = [(fillet with skin weight (g)/body weight (g)] × 100(5)

Hepatosomatic Index, HSI (%) = [(liver weight (g)/total body weight (g)] × 100(6)

Viscerosomatic Index, VSI (%) = [(viscera weight (g)/total body weight (g)] × 100(7)

Color measurements were performed on skin and fillet muscle in triplicate positions (cranial, medial, and caudal) with a CHROMA METER CR-200 (Konica Minolta, Chiyoda, Japan). The color was expressed as lightness (L*), redness index (a*), and yellowness index (b*), according to the CIELab system [30]. The mean values measured on the three positions (cranial, medial, and caudal) of the skin and fillet were used for data analysis. Muscle pH was measured at the cranial, medial, and caudal positions of the fillets with a SevenGo SG2™ pH-meter (Mettler-Toledo, Schwerzenbach, Switzerland) equipped with an Inlab puncture electrode (Mettler-Toledo, Ltd.).

Fillet texture analysis was performed using a Warner-Bratzler shear blade (width of 7 cm) with a Zwick Roell^®^ 109 texturometer (Zwick Roell, Ulm, Germany), equipped with a 1 kN load cell, setting the crosshead speed at 30 mm min^−1^. A section of 3 × 3 cm was cut from the epaxial cranial region of both fillets of each fish and then subjected to the force of the blade probe. Zwick Roell^®^ Test-Xpert2 3.0 software was used for texture data collection and analysis.

Afterward, fillets were skinned, homogenized, and used to determine the water-holding capacity (WHC) and chemical composition of fillet muscle, as follows. WHC was determined according to Iaconisi et al. [31] by calculating the amount of water retained by 2 g of sample after centrifugation (1500 rpm for 5 min). For each sample, WHC was performed in duplicate, and the mean value was used for data analysis.

### 2.5. Fillet Chemical Composition, Estimation of Indices of Elongase and Desaturase Activity and Oxidative Status

Moisture, crude protein (N × 6.25), and ash contents of skinned fillets were determined following AOAC methods [32]. The total lipid content of the fillets was determined after extraction performed according to Folch et al. [33]. The fatty acids (FAs) of the lipid extract were determined after transesterification to methyl esters (FAME) using a base-catalyzed transesterification [34]. The FA profile was determined by gas-chromatography using a Varian GC 430 gas chromatograph (Varian Inc., Palo Alto, CA, USA), equipped with a flame ionization detector and a Supelco Omegawax™ 320 m capillary column (Supelco, Bellefonte, PA, USA). Chromatograms were recorded using the Galaxie Chromatography Data System 1.9.302.952 (Varian Inc., Palo Alto, CA, USA). FAs were identified by comparing the FAME retention time with those of the Supelco 37 component FAME mix standard (Supelco, Bellefonte, PA, USA) and quantified through calibration curves, using tricosanoic acid (C23:0) (Supelco, Bellefonte, PA, USA) as internal standard.

To estimate the indices of elongase and desaturase activity of FAs, the ratio of the product/s to the precursor/s was calculated, as described by Bruni et al. [35], based on fillets FA composition. The following equations were used:

Thioesterase = C16:0/C14:0(8)

Elongase = C18:0/C16:0(9)

Δ9 desaturase (16) = [(C16:1n-9)/(C16:1 + C16:0)] × 100(10)

Δ9 desaturase (18) = [(C18:1n-9)/(C18:1 + C18:0)] × 100(11)

Δ9 desaturase (16 + 18) = [(C16:1 + C18:1)/(C16:1 + C16:0 + C18:1 + C18:0)] × 100(12)

Δ5 + Δ6 desaturase (n-6) = [(C20:2n-6 + C20:4n-6)/(C18:2n-6 + C20:2n.6 + C20:4n-6)] × 100(13)

Δ5 + Δ6 desaturase (n-3) = [(C20:5n-3 + C22:5n-3 + C22:6n-3/C18:3n-3 + C20:5n-3 + C22:5n-3 + C22:6n-3)] × 100(14)

The oxidative status of the fillets was determined by quantification of the conjugated dienes (CD) in 0.5 µL of lipid extract dissolved in 3 mL of pure hexane, according to Srinivasan et al. [36]. Secondary oxidative products were quantified in the livers and fillets as thiobarbituric acid reactive substances (TBARS) following the methods described by Pérez-Jiménez et al. [37] and Secci et al. [38], respectively. The results are expressed as mmol hydroperoxides (mmol Hp/100 g fillet) and malondialdehyde equivalents (mg MDA-eq/100 g fillet and, for liver, in nmol MDA-eq/g tissue) for CD and TBARS, respectively.

### 2.6. Hepatic Enzymatic Activity

Nine liver samples per treatment (*n* = 9) were homogenized (1:4) in ice-cold buffer (100 mM Tris-HCL buffer, containing 0.1 mM EDTA and 0.1% Triton X-100 (*v*/*v*); pH 7.8), and centrifuged at 30,000× *g* for 30 min at 4 °C. Then, supernatant was collected, divided into several aliquots, and stored at −80 °C for measurement of the key enzymes of the oxidative stress and of intermediary metabolism. Glutathione reductase (GR; EC 1.6.4.2), catalase (CAT; EC 1.11.1.6), and glutathione peroxidase (GPX; EC 1.11.1.9) activities were determined as previously described [39]. Key enzymes of the intermediary metabolism, including glucose-6-phosphate dehydrogenase (G6PDH; EC 1.1.1.49), malic enzyme (ME; EC 1.1.1.40), glutamate dehydrogenase (GDH; EC 1.4.1.2), and 3-hydroxyacyl-CoA dehydrogenase (HOAD; EC 1.1.1.35) activities were measured as described by Coutinho et al. [40]. Aspartate aminotransferase (AST/GOT; EC 2.6.1.1) and alanine aminotransferase (ALT/GPT; EC 2.6.1.2) activities were performed using commercial kits (Spinreact, AST/GOT; 41,273; ALT/GPT; 41,283). Total soluble proteins were determined according to Bradford [41] using bovine serum albumin solution as standard. All enzymatic assays were performed at 25 °C, except for alanine and aspartate aminotransferase activities that were carried out at 37 °C. Changes in absorbance were monitored with Multiskan Go microplate Spectrophotometer (Model 5111 9200; Thermo Scientific, Nanjing, China). Except for CAT, which is expressed as units per mg of soluble protein, the activities of the other enzymes are expressed as milliunits per mg of soluble protein. One unit of the enzyme was defined as the amount of enzyme required to convert 1 µmol of substrate per min under the assay conditions.

### 2.7. Statistical Analysis of Data

Values are expressed as mean ± standard deviation for data of the time before the experimental feeding (T0). The other data were subjected to one-way analysis of variance (ANOVA) using the PROC GLM of SAS/STAT Software, Version 9.4 [42]. A *p*-value of 0.05 was set as the minimum level of significance. Results are presented as LSM ± SEM.

## 3. Results

### 3.1. Growth Performance

European seabass fed on commercial and experimental diets showed adequate growth performance during the trial (Table 2). Fish survival was 98.8% and 98.7% in the CG and SSH groups, respectively. The higher, although not significant, weight and SGR found in fish fed the SSH diet could be related to the better FCR of this group compared with the CG group (1.40 vs. 1.60). The FI was 3.27 in both groups.

### 3.2. Marketable Characteristics of Fish and Physical Characteristics of Fillet

The total lipid content, the details of the FA profile, and the oxidative status of fillets at T0 are shown in Supplementary Table S4. After 66 days of feeding, the marketable traits of European seabass and the physical characteristics of fillets were not significantly affected by the dietary treatments, except for the skin color of the SSH, whose lightness (L*) was lower than that of the CG fish (*p* < 0.01) (Table 3). 

### 3.3. Fillet Chemical Composition, Estimation of Indices of Elongase and Desaturase Activity and Oxidative Status

The proximate composition of the fillets did not differ between the dietary groups (*p* > 0.05) (Table 4). An effect on the FA profile was observed (Table 4); in fact, the fillet contents of C18:4n-3 and C22:1n-11 were higher in CG fish (*p* < 0.05). Additionally, the total saturated fatty acids (SFA) content was not affected by the diet (*p* > 0.05), except for lauric acid (C12:0), which was significantly higher (*p* < 0.0001) in the SSH group (13.2 ± 0.73 and 1.70 ± 1.02 mg of FA/100 g fresh tissue in the SSH and CG groups, respectively). The primary (CD) and secondary (TBARS) oxidation products of the European seabass fillets were not affected by the dietary treatments (Table 4).

The indices of the lipid metabolism showed that elongase, Δ9 desaturase (C16), Δ9 desaturase (C18), and Δ9 desaturase (C16 + C18) activities were the highest in SSH fish (*p* < 0.05) (Table 5). Regarding the estimated activities of Δ5 + Δ6 desaturase n-3 activities, the CG group showed the highest values (*p* < 0.0001).

### 3.4. Enzymatic Activities

Hepatic CAT, GPX, GR, GDH, ME, and ALT activities, as well as lipid peroxidation, were not affected by the dietary treatments, while AST, HOAD, and G6PDH activities were the highest in fish fed the SSH diet (*p* < 0.05; Table 6).

## 4. Discussion

Aquaculture is striving toward the circular economy concept in its production process, and the path to sustainable, nutritious, and nonconventional aquafeed ingredients has been extensively investigated in controlled trials over the last few decades. However, little is known about research conducted under routine commercial farming conditions.

In the present study, after 66 days of feeding in a commercial farm, moderately higher growth and better zootechnical indices (K, SGR, FCR), although not statistically significant, were observed in fish fed the SSH diet. The eviscerated weight of SSH fish was also about 10% higher than that of fish fed CG diet, resulting in relevant commercial implications. No effect on growth performance was noticed in previous experimental studies in which European seabass were fed diets containing 19.5% HIM [18], or gilthead seabream fed diets containing 32.4% HIM and 27.5% PBM [6,24,25]. Thus, the present results confirm the possible use of HIM and PBM in a plant-rich diet for marine species previously observed on an experimental scale [24,25]. Aligning with the aforementioned findings, it was shown that European seabass fed the SSH diet improved its FCR, as reported in gilthead sea bream when fed a similar diet that was previously tested in an experimental setting [25], supported by the fact that a partial replacement of the plant mixture with HIM and PBM could also activate brush border membrane enzymes [26].

In the present study, the skin lightness (L*) of fish fed the SSH diet was lower than that of fish fed the commercial diet, but the relative difference between the two values was subtle. Similarly, the inclusion of PBM in a vegetable-based diet was not able to pigment the skin of gilthead seabream [43]. Future studies on consumer preferences for fish with different skin colors are envisaged to clarify whether changes such as those found in this study are perceived positively or negatively.

Diet can significantly impact fillet FA composition. In the present study, the sum of eicosapentaenoic (EPA) and docosahexaenoic (DHA) acid contents indicated that, independently of the diet, one serving portion of the fillets of this trial (150 g) would provide the consumer with 419.27 mg of EPA and DHA, an amount above the recommended daily intake [44]. On the other hand, the fillets of fish fed the SSH diet had higher levels of lauric acid (C12:0), most likely originating from the dietary inclusion of HIM. It has been widely reported that this insect species has a specific ability to convert other FAs into C12:0 [45], resulting in particularly high levels of this SFA, which may also impair the overall nutritional quality of fish fillets. However, previous studies have demonstrated that the inclusion of HIM up to 25%, 40%, or 50% of the total protein content in the diets for gilthead seabream and Siberian sturgeon, respectively, is associated with beneficial effects on fish gut health, such as immunostimulation and anti-inflammation [6,46]. These results are mainly attributed to the presence of bioactive compounds, including medium-short chain FAs, such as lauric acid [6].

Marine fish are known to have minimal desaturase activity; nonetheless, gilthead seabream was proven to express a desaturase gene [47,48]. Based on the present study, it could be assumed that the diets modulated the estimated indices of the FA elongase and desaturase activities. The higher MUFA desaturase values in the SSH group hint that the estimated desaturase activity on MUFAs was higher in the SSH than in the CG, while it seemed that the SSH fish produced n-3 FAs to a lesser extent than the CG. In all probability, this is a direct consequence of the fact that C18:3n-3 content was higher, and EPA and DHA contents were lower in the CG diet in comparison with the SSH, stimulating the fish to elongate and desaturate C18:3n-3 to EPA and DHA. Besides, the Δ5 + Δ6 desaturase n-3 index of the CG fish was higher, suggesting that this group needed to produce n-3 FAs endogenously.

Liver plays a key role in the metabolism of nutrients in fish, and a wide range of enzymatic antioxidants protect against pro-oxidant species, such as reactive oxygen species. It is a fact that when investigating the use of new ingredients or searching for aquafeed formulations, an alteration in hepatic metabolic activities and liver oxidative status can be observed [49], thus potentially indicating a health impairment. In the present study, there was an increased activity of G6PDH and HOAD in SSH fish, suggesting an increased β-oxidation and consequently increased utilization of FAs for energetic purposes. This could explain the significant reduction in C22:1n-11 fillet content in SSH fish despite its higher level in SSH feed. As verified by several authors, C22:1n-11 is largely used as a substrate for β-oxidation and is generally oxidized rather than stored in the body [50,51,52,53].

Another indicator of altered energy metabolism is the significant increase in AST in SSH fish. This enzyme, found in fish hearts, skeletal muscles, kidneys, and brains, assists in the transfer of the amino group from aspartic acid to α-ketoglutaric acid to form oxaloacetic and glutamic acids [49,54]. This pathway is well known in fish, and it is considered to be of paramount importance to maintain glucose homeostasis during periods of food deprivation [55]; it is generally considered to be a good indicator of the utilization of amino acids as an energy source [56]. In addition, as recently observed by [57] in Chinese sturgeon (*Acipenser sinensis*), AST amount in the liver increased as the specific growth and feeding rates increased. This could support the higher body weight (*p* > 0.05) and lower FCR (*p* > 0.05) of European seabass fed SSH, suggesting a better use of FAs and amino acids as energy sources. The factors determining this moderately positive effect remain unclear, even if the changes in the gut microbiome observed in diets containing HIM [19] underline that this ingredient is able to increase the abundance of two interesting taxa in fish, such as Bacillaceae and Paenibacillaceae, involved in the production of short-chain FAs and other useful molecules able to improve fish health [19].

While serum AST is frequently correlated with fish health [58], the same increase was not observed in serum, gills, liver, and other tissues following toxicant exposure [59]. This suggests that liver AST activity cannot be a reliable indicator of a diseased or stressful condition, which can induce oxidative stress in fish. The present study showed that the oxidative status of both fish liver and fillets was equivalent between the two dietary groups, in agreement with what was observed in rainbow trout fed diets containing HIM [60]. A previous study performed on European seabass demonstrated that a dietary inclusion of 6.5 and 13 g/100 g of HIM decreased liver oxidative stress [18], which was attributed to the presence of chitin. Indeed, chitin and its derivatives have been shown to act as antioxidants and prevent ROS formation in fish [61]. Furthermore, in this study, the negative effect of dietary PBM on the activity of antioxidant enzymes was not observed, contrary to what was previously reported on barramundi *(Lates calcarifer)* but at much higher levels, corresponding to the total replacement of FM [62].

## 5. Conclusions

The transition to new protein sources is a strategic need for more sustainable aquaculture. The present research tested for the first time the combined use of HIM and PBM as alternative and nutritious ingredients in diets for European seabass farmed under commercial conditions. The results show that feeding commercial-size European seabass with the new formulation for 66 days did not impair fish growth, oxidative stress response, fish marketability traits, or fillet quality characteristics. The formulated innovative diet, although poor in FM, showed comparable performance to the commercial aquafeed, demonstrating its practical utilization under the commercial farming conditions tested. In addition, the fillet EPA and DHA contents of fillets from fish fed diets including HIM and PBM can provide the recommended daily intake if one serving of the fish fillet is eaten. Nevertheless, long-term studies assessing the effects of incorporating *H. illucens* larvae and PBM in aquafeeds into commercial-scale production systems are essential to validate the present observations; in addition, exploring the possible utilization of the new formulation in the diet of other marine species should be encouraged.

## Figures and Tables

**Table 1:** Ingredient and proximate composition (% as fed) of the commercial (CG) and experimental (SSH) diets.

	CG ^§^	SSH
**Ingredient composition**		
Feeding stimulants ^1^		5.5
Veg.-protein mix ^2^		35.4
*Hermetia* meal ^3^		8.1
PBM ^4^		20.6
Wheat meal *		5.5
Whole pea *		8.8
Fish oil ^5^		6.2
Veg. oil mix ^6^		7.4
Vit. & Min. Premix ^7^		0.3
Choline HCL		0.1
Sodium phosphate		0.2
L-Lysine ^8^		0.1
DL-Methionine ^9^		0.3
Celite		1.5
**Chemical composition**		
Crude protein	45	45
Crude fat	18	20
Crude cellulose	1.3	1.8
Ashes	8.6	8
Calcium	1.6	1.7
Phosphorus	1.2	1.15
Sodium	0.3	0.2
Chitin		0.39
Gross Energy (MJ/kg)	20.1	20.3
P/E ratio	22.4	22.2

^§^ please see Table S1. ^1^ Feeding stimulants, g/100 diet: fish protein concentrate CPSP90-Sopropeche, France (CP: 82.6%), 3.5; Squid meal (CP: 80.3%), 2.0. ^2^ Vegetable–protein sources mixture (% composition): dehulled, toasted soybean meal, 39; soy protein concentrate-Soycomil, 20; maize gluten, 18; wheat gluten, 15; rapeseed meal, 8. ^3^ ProteinX™, Protix, Dongen, The Netherlands (CP: 55.4%; CF: 20.8% as fed). ^4^ Poultry by-product meal from Azienda Agricola Tre Valli; Verona, Italy (CP: 65.6%; CF: 14.8% as fed). ^5^ Fish oil: Sopropêche, Boulogne sur Mer, France. ^6^ Vegetable oil mixture (% composition): rapeseed oil, 56; linseed oil, 26; palm oil, 18. ^7^ Vitamin and mineral supplement (per kg of premix): Vit. A, 2,000,000 IU; Vit. D3, 200,000 IU; Vit. E, 30,000 mg; Vit. K3, 2500 mg; Vit. B1, 3000 mg; Vit. B2, 3000 mg; Vit. B3, 20,000 mg; Vit. B5, 10,000 mg; Vit. B6, 2000 mg; Vit. B9, 1500 mg; Vit. B12, 10 mg; Biotin, 300 mg; Stay C^®^, 90,000 mg; Inositol, 200,000 mg; Cu, 900 mg; Fe, 6000 mg; I, 400 mg; Se, 40 mg; Zn, 7500 mg. ^8^ L-lysine: 99% from Ajinomoto EUROLYSINE S.A.S, France. ^9^ DL-Methionine: 99% from EVONIK Nutrition & Care GmbH, Germany. * Wherever not specified, the ingredients composing the diets were obtained from Veronesi.

**Table 2:** Growth performance of European seabass fed the commercial (CG) or experimental (SSH) diets for 66 days.

Growth Parameters ^1^	CG	SSH	*p*-Value ^2^
BW (g)	441.7 ± 58.6	461.5 ± 74.8	ns
TL (cm)	32.9 ± 1.39	33.3 ± 1.70	ns
SL (cm)	28.6 ± 1.28	28.8 ± 2.06	ns
K (%)	1.24 ± 0.09	1.25 ± 0.09	ns
SGR	0.57 ± 0.21	0.63 ± 0.26	ns

^1^ BW, total body weight; TL, total length; SL, standard length; K, condition factor; SGR, specific growth rate. Values are reported as mean ± standard deviation. ^2^ ns, not significant (*p* > 0.05).

**Table 3:** Marketable characteristics of fish and physical characteristics of European seabass fillets before (T0) and after 66 days of feeding with commercial (CG) or experimental (SSH) diets.

Items ^1^	T0 ^2^	CG	SSH	*p*-Value ^3^
Eviscerated weight, g	263 ± 47.81	384.93 ± 18.0	421.06 ± 12.73	ns
FY, %	54.67 ± 2.09	55.85 ± 0.55	56.62 ± 0.39	ns
VSI, %	9.77 ± 1.36	11.11 ± 0.44	10.89 ± 0.31	ns
HSI, %	1.54 ± 0.44	2.33 ± 0.12	2.03 ± 0.09	ns
pH	6.36 ± 0.07	6.30 ± 0.02	6.31 ± 0.02	ns
Texture, N	72.40 ± 12.71	89.98 ± 6.44	89.10 ± 4.55	ns
WHC, %	97.27 ± 0.61	95.07 ± 0.76	93.84 ± 0.54	ns
**Skin colour**				
L*	43.91 ± 2.17	52.51 ± 0.73	49.72 ± 0.51	0.004
a*	−1.22 ± 0.29	−1.34 ± 0.17	−1.02 ± 0.12	ns
b*	0.61 ± 0.97	−0.55 ± 0.26	−0.57 ± 0.18	ns
**Fillet colour**				
L*	50.31 ± 0.83	49.18 ± 0.36	49.06 ± 0.25	ns
a*	−0.04 ± 0.60	−0.61 ± 0.20	−0.62 ± 0.14	ns
b*	0.92 ± 0.76	−1.03 ± 0.23	−0.84 ± 0.16	ns

^1^ FY: fillet yield; VSI: viscerosomatic index; HSI: hepatosomatic index; WHC: water holding capacity. ^2^ Values reported as mean ± standard deviation of triplicate analyses. ^3^ ns, not significant (*p* > 0.05).

**Table 4:** Chemical composition, fatty acid profile, and oxidative status of fresh fillets from European seabass fed the commercial (CG) or experimental (SSH) diets.

	CG	SSH	*p*-Value ^1^
**Proximate composition, g/100 g fresh tissue**			
Moisture	71.01 ± 0.47	71.02 ± 0.33	ns
Crude protein	20.05 ± 0.22	20.01 ± 0.15	ns
Ashes	1.03 ± 0.05	1.01 ± 0.03	ns
Total lipids	7.91 ± 0.50	7.96 ± 0.35	ns
**Fatty acids ^2^, mg of FA/100 g fresh tissue**			
C14:0	109.25 ± 7.90	114.18 ± 5.58	ns
C16:0	657.87 ± 47.86	662.18 ± 33.84	ns
C16:1n-7	158.77 ± 11.44	155.35 ± 8.08	ns
C18:0	133.77 ± 9.99	140.55 ± 7.07	ns
C18:1n-9	976.03 ± 80.81	1089.27 ± 57.14	ns
C18:1n-7	93.51 ± 6.88	98.24 ± 4.86	ns
C18:2n-6	542.68 ± 41.42	572.75 ± 29.29	ns
C18:3n-3	93.78 ± 7.17	104.23 ± 5.07	ns
C18:4n-3	36.40 ± 2.30	29.92 ± 1.63	0.030
C20:1n-9	82.90 ± 6.00	86.72 ± 4.24	ns
C20:5n-3	179.17 ± 10.79	164.18 ± 7.63	ns
C22:1n-11	55.03 ± 3.34	44.55 ± 2.36	0.016
C22:6n-3	258.37 ± 13.48	236.81 ± 9.53	ns
EPA + DHA	437.53 ± 24.21	400.99 ± 17.12	ns
ΣSFA	940.80 ± 68.46	969.14 ± 48.40	ns
ΣMUFA	1415.72 ± 111.16	1523.05 ± 78.60	ns
Σn-6 PUFA	602.51 ± 45.15	634.80 ± 31.92	ns
Σn-3 PUFA	613.45 ± 36.06	582.95 ± 25.50	ns
**Oxidative status ^3^**			
CD, µmol Hp/100 g fresh tissue	0.21 ± 0.01	0.22 ± 0.008	ns
TBARS, mg MDA-eq/100 g fresh tissue	0.02 ± 0.001	0.03 ± 0.001	ns

^1^ ns, not significant (*p* > 0.05). ^2^ SFA: saturated fatty acids; MUFA: monounsaturated fatty acids; PUFA: polyunsaturated fatty acids. The following fatty acids, below 1% of total FAME, were utilized for calculating the Σ classes of fatty acids, but they are not listed in the table: C12:0, C13:0, C14:1n-5, C15:0, C16:1n-9, C16:3n-4, C16:2n-4, C17:0, C17:1, C16:4n-1, C18:2n-4, C18:3n-6, C18:3n-4, C18:4n-1, C20:0, C20:1n-11, C20:1n-7, C20:2n-6, C20:3n-6, C20:4n-6, C20:3n-3, C20:4n-3, C22:0, C22:1n-9, C22:1n-7, C22:2n-6, C21:5n-3, C22:4n-6, C22:5n-6, C22:5n-3, C24:0, C24:1n-9. ^3^ CD, conjugated dienes; TBARS, thiobarbituric acid reactive substances.

**Table 5:** Estimated indices of FAs elongase and desaturase activity in fresh fillets from European seabass fed the commercial (CG) or experimental (SSH) diets after a 66-day feeding trial.

	CG	SSH	*p*-Value ^1^
Thioesterase	6.03 ± 0.10	5.80 ± 0.07	ns
Elongase	0.20 ± 0.003	0.21 ± 0.002	0.028
Δ9 desaturase (C16)	59.65 ± 0.24	62.13 ± 0.17	<0.0001
Δ9 desaturase (C18)	87.88 ± 0.19	88.53 ± 0.13	0.011
Δ9 desaturase (C16 + C18)	55.50 ± 0.26	57.86 ± 0.18	<0.0001
Δ5 + Δ6 desaturase n-6	6.87 ± 0.18	6.79 ± 0.13	ns
Δ5 + Δ6 desaturase n-3	83.16 ± 0.48	80.54 ± 0.34	0.0001

^1^ ns, not significant (*p* > 0.05).

**Table 6:** Activities of the hepatic intermediary metabolism enzymes and of the antioxidant enzymes (mU/mg protein) and lipid peroxidation (nmol MDA-eq/g tissue) of European seabass fed the commercial (CG) or experimental (SSH) diets.

	CG	SSH	*p*-Value ^1^
**Intermediary metabolism enzymes ^2^**			
GDH	69.29 ± 5.04	77.38 ± 5.04	ns
ALT	35.59 ± 2.76	38.36 ± 2.76	ns
AST	24.29 ± 2.18	34.38 ± 2.31	0.01
ME	6.50 ± 0.57	6.58 ± 0.57	ns
HOAD	8.23 ± 0.81	11.32 ± 0.81	0.01
**Antioxidant enzymes ^3^**			
CAT	22.25 ± 2.03	23.61 ± 2.03	ns
G6PDH	328.97 ± 29.04	493.60 ± 29.04	0.001
GPX	17.75 ± 1.72	19.19 ± 1.72	ns
GR	4.11 ± 0.42	3.49 ± 0.40	ns
**Liver lipid peroxidation ^4^**			
LPO	13.99 ± 1.32	13.17 ± 1.38	ns

^1^ ns, not significant (*p* > 0.05). ^2^ GDH: glutamate dehydrogenase; ALT: alanine aminotransferase; AST: aspartate aminotransferase; ME: malic enzyme; HOAD: 3-hydroxyacyl CoA dehydrogenase. ^3^ CAT: catalase; G6PDH: glucose 6-phosphate dehydrogenase; GPX: glutathione peroxidase; GR: glutathione reductase. ^4^ LPO: lipid peroxidation.

## Data Availability

The original contributions presented in the study are included in the article/supplementary material, further inquiries can be directed to the corresponding author/s.

## Supplementary Materials

The following supporting information can be downloaded at https://www.mdpi.com/article/10.3390/ani14101449/s1, Table S1: Proximate composition, chitin (g/100 g), and gross energy contents of the partially defatted *Hermetia illucens* meal; Table S2: Ingredient composition of the commercial diet; Table S3: Fatty acid profile (% of the total fatty acid methyl esters, FAMEs); Table S4: Mean ± dev.st. of total lipids (g/100 g), the contents of fatty acids profile (mg of FA/100 g of fresh tissue) and oxidative status of fillets from *Dicentrarchus labrax* at the beginning of the trial (T0).
