# Supplementary material for: Growth, Hepatic Enzymatic Activity, and Quality of European Seabass Fed on Hermetia illucens and Poultry By-Product Meal in a Commercial Farm
Source: Animals (Basel). 2024 May 13;14(10):1449. doi: 10.3390/ani14101449 (PMC11117365; doi:10.3390/ani14101449)
Supplement: Supplementary file 1 [file animals-14-01449-s001.zip › animals-2974123-supplementary.pdf]

Table S1. Proximate composition, chitin (g/100g) and gross energy contents of the partially defatted *Hermetia illucens* meal.

	HIM
Moisture	4.4
Crude protein (N × 6.25)	55.4
Crude fat (Soxhlet)	20.8
Ashes	6.4
Crude fibre	10.3
Chitin	4.7
Gross Energy MJ/kg	20.76

Table S2. Ingredient composition of the commercial diet[1]

Fish meal, dehulled and toasted soybean meal, PAP[2] from poultry, wheat, maize gluten, fish oil, porcine-hemoglobine powder, soybean oil, hulled peas, wheat distillers, wheat gluten, monocalcium phosphate, whey powder.
Aditives:vitamin and mineral supplement, citric acid, propyl Gallate, BHT[3]

[1] Note that the producer did not provide the % of each ingredient.
[2]PAP, processed animal product.
[3]BHT, *butylated hydroxytoluene.*

Table S3. Fatty acid profile (% of the total fatty acid methyl esters, FAMEs).

	**CG**	**SSH**
**Fatty acid profile**[1]		
C12:0	0.10 ± 0.05	2.43 ± 0.45
C14:0	1.29 ± 0.11	3.95 ± 0.37
C16:0	14.79 ± 0.54	14.82 ± 0.38
C16:1n-7	1.98 ± 0.19	4.25 ± 0.20
C18:0	4.69 ± 0.04	3.82 ± 0.13
C18:1n-9	26.72 ± 0.46	30.22 ± 0.80
C18:1n-7	2.15 ± 0.12	2.83 ± 0.14
C18:2n-6	32.62 ± 0.45	14.32 ± 0.67
C18:3n-3	5.33 ± 0.01	3.41 ± 0.01
C20:1n-9	1.11 ± 0.01	2.27 ± 0.01
C20:5n-3	1.21 ± 0.00	4.66 ± 0.02
C22:1n-11	0.54 ± 0.01	1.27 ± 0.04
C22:6n-3	3.22 ± 0.02	5.19 ± 0.07
ΣSFA	*22.15 ± 0.72*	*26.40 ± 1.11*
ΣMUFA	*33.21 ± 0.43*	*41.79 ± 0.82*
Σn-6 PUFA	*33.79 ± 0.52*	*15.73 ± 1.18*
Σn-3PUFA	*10.61 ± 0.17*	*15.05 ± 0.73*

[1] Values reported as mean ± standard deviation of triplicate analyses. SFA: saturated fatty acids; MUFA: monounsaturated fatty acids; PUFA: polyunsaturated fatty acids. The following fatty acids, below 1% of total FAME, were utilised for calculating the Σ classes of fatty acids but they are not listed in the table: C13:0, C14:1n-5, isoC15:0, C15:0, C16:1n-9, C16:2n-4, C17:0, C16:3n-4, C17:1, C16:4n-1, C18:2n-4, C18:3n-6, C18:3n-4, C18:4n-3, C18:4n-1, C20:0, C20:1n-11, C20:1n-7, C20:2n-6, C20:3n-6, C20:4n-6, C20:3n-3, C20:4n-3, C22:0, C22:1n-9, C22:1n-7, C22:2n-6, C21:5n-3, C22:4n-6, C22:5n-6, C22:5n-3, C24:0, C24:1n-9

Table S4. Mean ± dev.st. of total lipids (g/100 g), fatty acids (FA) profile contents (mg of FA/100 g of fresh tissue) and oxidative status of fillets from *Dicentrarchus labrax* at the beginning of the trial (T0).

	**T0**
**Total lipids**	7.41 ± 1.16
**Fatty acids**	
C14:0	89.80 ± 14.98
C16:0	631.86 ± 105.72
C16:1n-7	129.54 ± 21.57
C18:0	139.70 ± 26.41
C18:1n-9	983.46 ± 171.48
C18:1n-7	84.29 ± 14.53
C18:2n-6	589.61 ± 102.95
C18:3n-3	99.03 ± 16.52
C18:4n-3	28.76 ± 4.99
C20:1n-9	70.59 ± 12.08
C20:5n-3	136.25 ± 21.27
C22:1n-11	45.68 ± 8.18
C22:6n-3	212.42 ± 29.77
ΣSFA	*895.38 ± 151.48*
ΣMUFA	*1358.57 ± 233.92*
Σn-6 PUFA	*643.34 ± 110.69*
Σn-3 PUFA	*514.21 ± 78.59*
**Oxidative status**	
Conjugated dienes, mmol Hydroperoxides/100 g fresh tissue	0.19 ± 0.03
TBARS[1], mg MDA-eq/100 g fresh tissue	0.03 ± 0.006

[1]Thiobarbituric acid reactive substances

SFA: saturated fatty acids; MUFA: monounsaturated fatty acids; PUFA: polyunsaturated fatty acids. The following FAs (below 1% of total FAME) were utilised for calculating the Σ classes of FAs but they are not listed in the table: C12:0, C13:0, C14:1n-5, C15:0, C16:1n-9, C16:3n-4, C16:2n-4, C17:0, C17:1, C16:4n-1, C18:2n-4, C18:3n-6, C18:3n-4, C18:4n-1, C20:0, C20:1n-11, C20:1n-7, C20:2n-6, C20:3n-6, C20:4n-6, C20:3n-3, C20:4n-3, C22:0, C22:1n-9, C22:1n-7, C22:2n-6, C21:5n-3, C22:4n-6, C22:5n-6, C22:5n-3, C24:0, C24:1n-9.
